# Pretreatment neutrophil-to-lymphocyte ratio predicts prognosis in patients with diabetic macular edema treated with ranibizumab

**DOI:** 10.1186/s12886-019-1200-4

**Published:** 2019-08-27

**Authors:** Yuxiang Hu, Yi Cheng, Xiaoxuan Xu, Bo Yang, Feng Mei, Qiong Zhou, Li Yan, Jun Wang, Xiaorong Wu

**Affiliations:** 1 Department of Ophthalmology, the First Affiliated Hospital of Nanchang University, No. 17, Nanchang, 330006 China; 2 Department of Ophthalmology, Xinjiang People’s Hospital, Urumqi, China; 3 Second Department of Respiratory Disease, Jiangxi Provincial People’s Hospital, Nanchang, China

**Keywords:** Diabetic retinopathy, Macular edema, Neutrophil–lymphocyte ratio, Predictors, Prognosis

## Abstract

**Background:**

To investigate the prognostic value of the neutrophil-to-lymphocyte ratio (NLR) in patients with diabetic macular edema (DME) treated monthly with ranibizumab.

**Methods:**

We retrospectively analyzed the medical records of all patients who received intravitreal ranibizumab (IVR) treatment for DME at the First Affiliated Hospital of Nanchang University between December 2015 and December 2017. Clinicopathological parameters, including NLR, were evaluated to identify predictors of better outcomes of IVR monotherapy.

**Results:**

Ninety-one treatment-naïve eyes treated with IVR for DME were retrospectively analyzed in this study. Baseline best-corrected visual acuity (BCVA), neutrophils, NLR, monocyte-to-lymphocyte ratio, and platelet-to-lymphocyte ratio were negatively correlated with the changes in BCVA at 24 weeks compared with the baseline, while baseline central retinal thickness and lymphocytes were positively correlated with the changes in BCVA at 24 weeks compared with the baseline. Multiple linear regression analysis revealed that NLR was independently associated with the mean change of BCVA between baseline and week 24. In addition, patients with NLR < 2.27 showed a better improvement in letter score than those with NLR > 2.27.

**Conclusion:**

Pretreatment NLR is independently associated with the BCVA in DME patients treated with IVR, and higher pretreatment NLR may contribute to inferior BCVA outcomes.

## Background

Diabetic macular edema (DME) is one of the major causes of visual impairment and even blindness in diabetic patients, occurs at any stage of diabetic retinopathy (DR) [1]. Intravitreous injections of anti-vascular endothelial growth factor (anti-VEGF) agents remain the most popular first-line therapy for DME [2]. Ranibizumab was the first FAD-approved anti-VEGF agent that has been shown to be beneficial and relatively safe for the treatment of DME [3]. Although most DME patients improved their best-corrected visual acuity (BCVA) after monthly intravitreal ranibizumab (IVR) treatment, some DME patients’ post-IVR BCVA did not improve significantly or even got worse [4, 5]. The societal impact of DME-associated vision loss, coupled with the substantial burden of patients and the required regular delivery of intravitreal anti-VEGF treatments, makes it necessary to identify and better understand clinical markers that may predict and assess long-term treatment burdens of patients.

The pathogenesis of DR is very complex, and the disease is progressive. Many factors are involved in the pathogenesis of DR, including blood glucose and lipid metabolism disorder, inflammatory response mechanism, hemodynamic changes, oxidative stress response, cytokines, and the patients’ genes. Some studies suggest that chronic inflammation caused by disorders of glucose and lipid metabolism often damages the retinal capillaries and leads to retinopathy. Therefore, DR is also classified as a chronic inflammatory disease [6]. Neutrophil-to-lymphocyte ratio (NLR), monocyte-to-lymphocyte ratio (MLR), and platelet-to-lymphocyte ratio (PLR) have been proven to be potential inflammatory markers in various conditions, including tumors [7–10], cardiovascular diseases [11, 12], and other diseases [13]. Some studies have also reported that PLR and NLR are associated with diabetes and its complications [14]. Yu et al. showed that NLR was elevated both in type 2 diabetes mellitus (T2DM) and in DR and was independently associated with the brachial-ankle pulse wave velocity [15]. MLR was also found to be an independent risk factor for DR in patients with T2DM [16]. Therefore, we performed the present study to analyze the prognostic impact of baseline NLR, MLR, and PLR in DME patients treated with ranibizumab. We hypothesized that an early decline of NLR during ranibizumab treatment would indicate a more favorable prognosis independent of established prognostic factors at baseline and that an increase of NLR would be associated with the opposite effect.

## Methods

### Study population

We conducted a retrospective study of patients diagnosed with DME who received initial IVR monotherapy at the First Affiliated Hospital of Nanchang University, Nanchang, China between December 2015 and December 2017. This study was approved by the Medical Ethics Committee of the First Affiliated Hospital of Nanchang University and adhered to the Declaration of Helsinki. The clinical records of all consecutive patients with DME were reviewed. DME was diagnosed by clinical examination and confirmed by optical coherence tomography (OCT) and fundus fluorescein angiography (FFA). Patients who were initially diagnosed with DME and regularly followed up for at least 24 weeks were included in the study. Patients were excluded if their eyes had any of the following conditions: (1) occurrence of vitreous hemorrhage before IVR treatment; (2) other treatments for DME during the follow-up period, such as laser photocoagulation, vitrectomy, and intravitreal corticosteroid injection; (3) other concomitant ocular diseases such as posterior uveitis, age-related macular degeneration, or retinal vascular occlusion; (4) the systemic or topical application of non-steroidal anti-inflammatory drugs and steroid drugs; and (5) history of an acute coronary event or stroke in the previous 3 months. In patients who received IVR treatment in both eyes, only the eye first treated was analyzed. All patients provided informed consent for IVR treatment and for the use of their data for research purposes.

### IVR monotherapy and follow-up

All patients received three consecutive monthly intravitreal injections of 0.5 mg ranibizumab under aseptic conditions. Patients were examined 1 week after injection and then monthly for at least 6 months. BCVA, intraocular pressure, slit-lamp biomicroscopy, dilated fundus ophthalmoscopy, and OCT were performed at each visit. Injections may be resumed if OCT and fundus examination revealed significant macular edema or subretinal fluid, or the BCVA deteriorated within the follow-up period. All patients and their families were informed and signed informed consents before the IVR treatment.

### Clinical examination and biochemical analysis

All participants received routine examination and ophthalmology examination and asked in detail about their medical history and records before IVR treatment. BCVA was examined by using the Early Treatment Diabetic Retinopathy Study (ETDRS) standard charts. The initial visual acuity test was performed at a distance of four meters, allowing the patient to slowly read from the top of the eye chart (one letter per second). The supervisor indicated the correct letter with a circle mark on the record sheet, wrong letters with X, and added no mark if the patient did not respond to a letter. If the patient made two or more errors in a row, the test was stopped. If the patient read fewer than four letters at a distance of four meters, the test was performed at a smaller distance of 1 m by only reading the first six lines while increasing the + 0.75 spherical diopter to compensate for the shorter distance. OCT was performed to evaluate the central retinal thickness (CRT) using an OCT system (Cirrus HD–OCT; Carl Zeiss Meditec, Inc. Dublin, CA, USA). The scanned area was 6 × 6 mm, the mode was 512 × 128, the axial resolution was 5 μm, and the detection depth was 2 mm. The CRT value was automatically measured by the instrument, but also measured by manually adjusting the accurate positioning. All OCT exams were performed by the same qualified physician who was blinded for the patients’ vision when analyzing the OCT images.

The patients’ fasting peripheral blood was collected prior to the first IVR treatment. Complete blood cell counts with differential counts were performed at the baseline. NLR, MLR, and PLR were calculated for all blood samples. All biochemical analyses of the same samples were performed in our hospital, including the analyses of glycosylated hemoglobin A1c (HbA1c), total cholesterol (TC), triglyceride (TG), high-density lipoprotein cholesterol (HDL), low-density lipoprotein cholesterol (LDL), serum creatinine (Scr), and blood urea nitrogen (BUN).

### Statistical analysis

Continuous variables are expressed as mean ± standard deviation (SD) for normally distributed data and median (interquartile range [IQR]) for non-normally distributed data. Categorical variables are presented as number (percentage). Chi-square tests were used to determine associations between dichotomous variables. Pearson’s or Spearman’s correlation analysis was performed between basic parameters of the patients and their BCVA as well as CRT changes. To identify which factors are significantly associated with the BCVA change, univariate and multivariate linear analyses were performed using the stepwise method. Several parameters known as risk factors for BCVA change, including baseline BCVA, baseline CRT, lymphocytes, neutrophils, NLR, MLR, and PLR, were examined. Non-parametric analyses were used to compare the data. Statistical analyses were performed using the software SPSS 22.0 (SPSS, Inc., Chicago, IL, USA), and values of *p* < 0.05 were considered statistically significant.

## Results

The outcomes of 91 treatment-naïve eyes of 91 DME patients treated with IVR were retrospectively analyzed in this study. The clinical and biochemical characteristics of all patients are summarized in Table 1.

**Table 1:** Baseline characteristics of 91 DME patients

Characteristic	
Age (years)	54 [47–61]
Male sex (%)	57 (62.6)
Duration of DM (years)	6.00 [5.00–8.00]
SBP (mmHg)	135 [121–147]
DBP (mmHg)	83 [72–90]
HbA1c (%)	7.24 ± 1.56
TC (mmol/L)	5.06 ± 1.21
TG (mmol/L)	1.52 [0.94–2.03]
HDL (mmol/L)	1.15 [0.90–1.42]
LDL (mmol/L)	2.73 [2.24–3.39]
Scr	86.90 [62.70–117.00]
BUN	6.00 [5.10–8.30]
Mean BCVA (letter score)	45.99 ± 12.74
Mean CRT (μm)	484.65 ± 113.08
WBC (× 10^9^/L)	5.93 [4.88–7.48]
Neutrophils (×10^9^/L)	3.51 [2.73–4.84]
Monocytes (× 10^9^/L)	0.40 [0.30–0.48]
Platelets (× 10^9^/L)	186.00 [140.00–237.00]
Lymphocytes (× 10^9^/L)	1.62 [1.29–1.90]
NLR	2.27 [1.72–2.93]
MLR	0.24 [0.18–0.30]
PLR	114.37 [86.02–146.74]

Data are expressed as mean (SD), median (inter-quartile range), or percentage

*DM* diabetes mellitus, *SBP* systolic blood pressure, *DBP* diastolic blood pressure, *WBC* white blood cell, *HbA1c* Hemoglobin A1c, *TC* total cholesterol, *TG* triglycerides, *HDL* high-density lipoprotein cholesterol, *LDL* low-density lipoprotein cholesterol, *Scr* serum creatinine, *BUN* blood urea nitrogen, *BCVA* best-corrected visual acuity, *CRT* central retinal thickness, *WBC* white blood cell, *NLR* neutrophil-to-lymphocyte ratio, *MLR* monocyte-to-lymphocyte ratio, *PLR* platelet-to-lymphocyte ratio.

To identify factors that determine the therapeutic response to IVR at 24 weeks, correlations of changes in either BCVA or CRT between baseline and week 24 as well as baseline characteristics in individual patients were evaluated, as summarized in Table 2. Baseline BCVA, neutrophils, NLR, MLR, and PLR were negatively correlated with the changes in BCVA at 24 weeks compared with the baseline, while baseline CRT and lymphocytes were positively correlated with the changes in BCVA at 24 weeks compared with the baseline. No correlation was found between baseline clinical factors and changes in CRT at 24 weeks compared with the baseline.

**Table 2:** Association between baseline clinical factors and changes in either BCVA or CRT at week 24

	Change in BVCA		Change in CRT	
	Correlation coefficient	*P* value	Correlation coefficient	*P* value
Age	−0.063	0.555	−0.013	0.903
duration of DM	0.090	0.394	0.139	0.189
SBP	0.018	0.864	−0.146	0.166
DBP	−0.012	0.913	−0.018	0.867
HbA1c	0.021	0.845	0.138	0.191
TC	0.027	0.800	0.148	0.161
TG	0.027	0.797	0.044	0.681
HDL	−0.072	0.499	0.016	0.880
LDL	−0.085	0.426	0.078	0.462
Scr	0.157	0.137	−0.006	0.953
BUN	0.054	0.610	−0.089	0.401
Baseline BCVA	−0.305	0.003	0.046	0.662
Baseline CRT	0.275	0.008	0.170	0.107
WBC	−0.166	0.115	−0.037	0.729
Neutrophils	−0.383	< 0.001	0.061	0.564
Monocytes	−0.074	0.486	0.031	0.770
Platelets	−0.024	0.819	0.017	0.871
Lymphocytes	0.399	< 0.001	−0.062	0.559
NLR	−0.660	< 0.001	0.130	0.218
MLR	−0.392	< 0.001	0.058	0.585
PLR	−0.335	0.001	0.045	0.670

*DM* diabetes mellitus, *SBP* systolic blood pressure, *DBP* diastolic blood pressure, *WBC* white blood cell, *HbA1c* Hemoglobin A1c, *TC* total cholesterol, *TG* triglycerides, *HDL* high-density lipoprotein cholesterol, *LDL* low-density lipoprotein cholesterol, *Scr* serum creatinine, *BUN* blood urea nitrogen, *BCVA* best-corrected visual acuity, *CRT* central retinal thickness, *WBC* white blood cell, *NLR* neutrophil-to-lymphocyte ratio, *MLR* monocyte-to-lymphocyte ratio, *PLR* platelet-to-lymphocyte ratio.

The correlation between age, SBP, DBP, TG, HDL, LDL, Scr, BUN, WBC, neutrophils, monocytes, platelets, lymphocytes, NLR, MLR, PLR and BCVA change were analyzed by Spearman’s correlation analysis, while HbA1c, TC, baseline BCVA, baseline CRT were analyzed by Pearson’s correlation analysis.

**Fig. 1:**
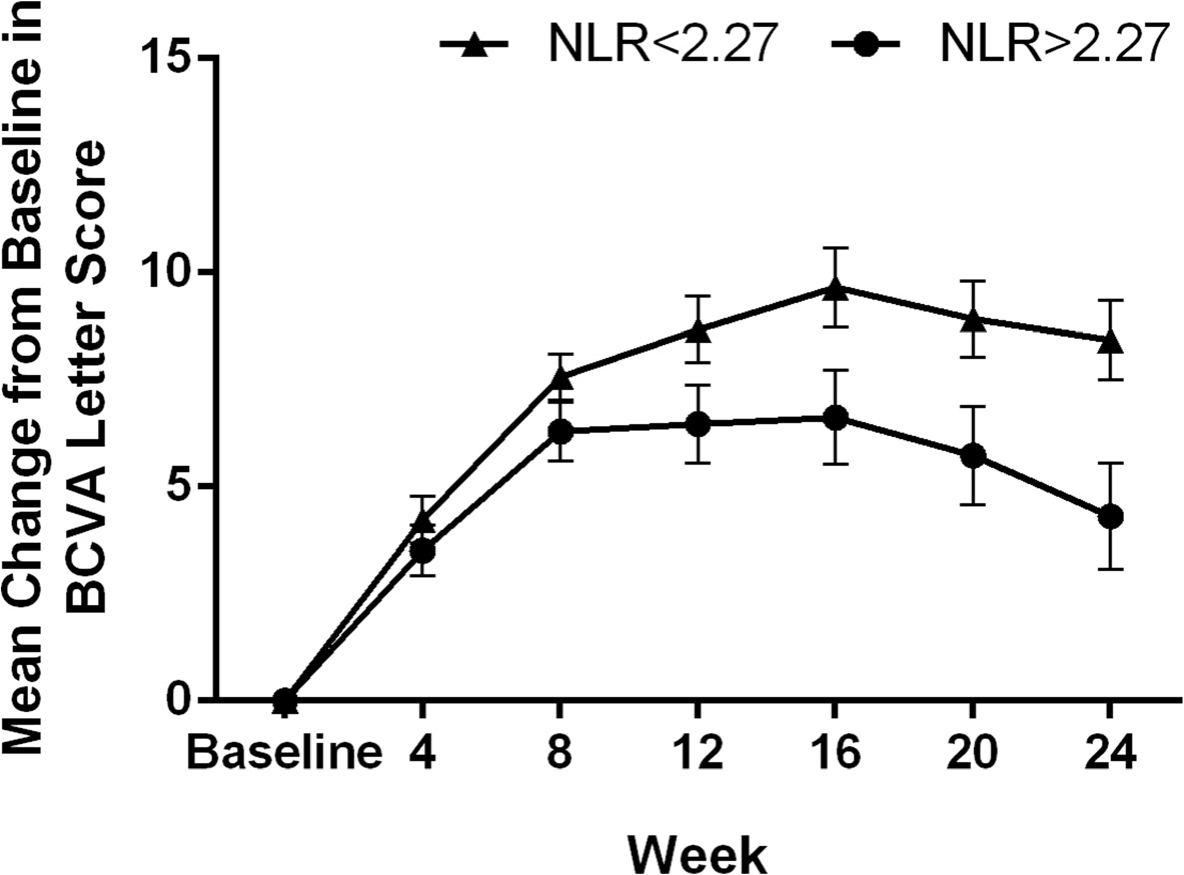
Mean changes in BCVA after intravitreal ranibizumab injection with respect to the baseline and at each follow-up in the two groups. Bars indicate 95% confidence intervals. *NLR* neutrophil-to-lymphocyte ratio; *BCVA* best-corrected visual acuity

Univariate linear regression was used to analyze factors that were significantly associated with changes in BCVA based on correlation analysis in all patients (Table 3). Baseline BCVA, baseline CRT, lymphocytes, neutrophils, NLR, MLR, and PLR were independently associated with changes in BCVA at week 24. Multivariate analysis revealed that baseline BCVA (B = − 0.081, *p* = 0.003) and NLR (B = − 1.884, *p* < 0.001) were the only significant predictors of changes in BCVA in DME patients treated with IVR. Interestingly, only NLR was a significant factor associated with the changes in BCVA, while the other two inflammatory predictors (MLR and PLR) were no significant factors. Thus, correlations between changes in BCVA at 24 weeks and baseline in different NLR groups were further evaluated. The group with NLR < 2.27 showed a greater improvement in the BCVA letter score than the group with NLR > 2.27 at 8, 12, 16, 20, and 24 weeks (*p* = 0.004, < 0.001, < 0.001, < 0.001, and < 0.001, respectively; Fig. 1). However, no significant difference between the two groups was found at 4 weeks.

**Table 3:** Univariate and multivariate linear regression analyses for predicting BCVA changes in DME patients treated with IVR

	B	SE	β	T	*P* value
Univariate linear regression					
Baseline BCVA	−0.105	0.033	−0.319	− 3.177	0.002
Baseline CRT	0.010	0.004	0.280	2.753	0.007
Lymphocytes	3.049	0.812	0.370	3.756	<0.001
Neutrophils	−0.983	0.237	−0.403	−4.151	<0.001
NLR	−1.986	0.276	−0.607	−7.202	<0.001
MLR	−15.471	3.929	−0.385	−3.938	<0.001
PLR	−0.035	0.009	−0.387	− 3.955	<0.001
Multivariate linear regression					
Baseline BCVA	−0.081	0.027	−0.246	−3.027	0.003
NLR	−1.884	0.266	−0.576	−7.079	<0.001

*SE* standard error, *BCVA* best-corrected visual acuity, *CRT* central retinal thickness, *NLR* neutrophil-to-lymphocyte ratio, *MLR* monocyte-to-lymphocyte ratio, *PLR* platelet-to-lymphocyte ratio

In multivariate linear regression using stepwise procedure, baseline BCVA and NLR were significant factors, whereas baseline CRT, lymphocytes, neutrophils, MLR, and PLR were excluded to decide final linear model.

## Discussion

Anti-VEGF drugs, such as ranibizumab, have been widely used in the clinical treatment of DME in recent years. Despite its superiority to laser photocoagulation, which was considered to be the first-line treatment before, less than 20% of the patients had no improvement in visual acuity or macular thickness after anti-VEGF therapies [17]. In this study, we found that the pretreatment NLR was independently associated with the mean change of BCVA between baseline and week 24 in DME patients who have been treated with IVR. In addition, patients with pretreatment NLR < 2.27 could have a better improvement in the letter score than those with pretreatment NLR > 2.27.

Increasing evidence shows that the inflammatory process plays a very important role in the pathogenesis of DR. A number of studies have shown that various systemic and localized (vitreous and aqueous) inflammatory cytokines are associated with the progression of DR [3]. As a manifestation of DR, DME arises from the intraretinal accumulation of fluid caused by blood-retina barrier (BRB) failure and augmented vascular permeability. In addition, DME patients exhibit a range of factors in the vitreous fluid, which are associated with inflammation; thus, inflammation may contribute to BRB failure and the subsequent development of DME [6]. Moreover, treatments targeting inflammatory factors could potentially prevent an increase in the vascular permeability in DME patients, suggesting that inflammation is really the key factor in the pathogenesis of DME [18]. Thus, DME has a close relationship to inflammation.

NLR is a marker of inflammation-associated alterations in peripheral blood leukocytes; thus, it has been comprehensively investigated as a potential indicator of systemic inflammation [19]. It has been shown to have a prognostic value for the prediction of the survival of cancers [20, 21], cardiovascular diseases [22], and intracerebral hemorrhage [23]. In the present study, we found that the pretreatment NLR was independently associated with the BCVA outcomes in DME patients after IVR treatment during a 24-week follow-up, and high NLR (> 2.27) causes more likely worse BCVA outcomes. Ulu et al. [24] showed that higher NLR can be used as an indicator of systemic inflammation in diabetic patients. Shiny et al. [25] also found that patients with T2DM showed a significantly higher NLR (2.2 ± 1.12) compared with impaired glucose tolerance subjects (1.82 ± 0.63). Another study also indicated that an elevated NLR (> 2.0) was significantly associated with insulin resistance in patients with T2DM [26]. The mechanism underlying the association between elevated NLR and worse outcomes is unclear. High NLR is associated with systemic inflammation, while a high level of neutrophils is considered to be a reservoir of vascular endothelial growth factor, which is critical in DME prognosis [27]. Therefore, NLR may be a significant prognostic factor for DME when patients are treated with ranibizumab or other anti-VEGF drugs.

As potential new inflammatory markers, MLR and PLR were also found to be closely associated with the progression and prognosis of many diseases, such as tumors [28, 29], *Plasmodium falciparum* malaria [30], cardiovascular diseases [31, 32], and retinal disease [13]. In this study, we also investigated the relationship between the BCVA at week 24 and the MLR and PLR at the baseline. Our results indicated that MLR and PLR were negatively correlated with the BCVA at week 24, but not associated with the BCVA at week 24 according to multiple linear regression analyses. A previous report suggested the PLR is an independent risk factor for early and late mortality in patients with DM [33]. Moreover, higher PLR is also related to the higher risk for 90-day incidence of readmission and mortality in patients with diabetic ketoacidosis [34]. In addition, Chen et al. [16] reported that the MLR is an independent risk factor for DR in Chinese patients with T2DM. The baseline MLR and PLR may also be related to the prognosis of DME patients treated with IVR, but this relation has not been confirmed in this study. However, this may also be due to the limitations of our research, and we will further explore and confirm this relation in future studies.

Importantly, baseline BCVA was also independently associated with an improved BCVA at 24 weeks in this study. Campochiaro et al. [35] found that DME patients with better BCVA at the baseline improved better after IVR treatment compared with those with worse BCVA at the baseline. Ozawa et al. [36] also reported similar results, suggesting that an early IVR treatment may be more efficient in DME patients. However, in the present study, an association between CRT values at the baseline and BCVA or CRT at 24 weeks has not been confirmed. In contrast, we found that better BCVA and CRT values at the end of the follow-up were associated with better CRT at baseline, which is not in agreement with the results of Ozawa et al. [36]. This may also be related to different follow-up times as well as individual diversities. Further studies are required to obtain a definitive conclusion.

There were some limitations in the present study. First, the study constituted a retrospective analysis that involved potential selection bias, although we included all consecutive patients who received IVR treatment during the study period. Second, most patients received treatment as part of an early access program; physicians involved in the study were therefore required to follow stringent inclusion and exclusion criteria with respect to IVR therapy. Importantly, the study thus excluded patients who had concomitant infections or were undergoing steroid treatment, which might have led to falsely elevated blood parameters. Third, the small number of patients and events in our cohort prevented comprehensive multivariable analyses and interfered with our ability to make definitive conclusions. Although it remains unclear whether NLR is prognostic or predictive in patients with DME who are undergoing IVR treatment, its low cost and ease of use warrant further evaluation of this marker.

## Conclusions

High pretreatment NLR is a simple prognostic marker strongly correlated with poor BCVA improvement in DME patients treated with IVR. NLR is a cheap and readily available biomarker adding additional prognostic information for the identification of patients benefiting from IVR treatment. Despite their limitations, our results may be useful for discussions with patients prior to immunotherapy. Larger prospective studies are necessary to confirm our findings.

## Data Availability

The datasets used and analyzed during the current study are available from the corresponding author on reasonable request.

## Abbreviations

BCVA: Baseline best-corrected visual acuity
BUN: Blood urea nitrogen
CRT: Central retinal thickness
CRT: Central retinal thickness
DBP: Diastolic blood pressure
DME: Diabetic macular edema
DR: Diabetic retinopathy
ETDRS: Early Treatment Diabetic Retinopathy Study
FFA: Fundus fluorescein angiography
HbA1c: Glycosylated hemoglobin A1c
HDL: High-density lipoprotein cholesterol
IQR: Interquartile range
IVR: Intravitreal ranibizumab
LDL: Low-density lipoprotein cholesterol
MLR: Monocyte-to-lymphocyte ratio
NLR: Neutrophil-to-lymphocyte ratio
OCT: Optical coherence tomography
PLR: Platelet-to-lymphocyte ratio
SBP: Systolic blood pressure
Scr: Serum creatinine
SD: Standard deviation
T2DM: Type 2 diabetes mellitus
TC: Total cholesterol
TG: Triglyceride
VEGF: Vascular endothelial growth factor
WBC: White blood cell
